# The alveolar epithelial cells are involved in pulmonary vascular remodeling and constriction of hypoxic pulmonary hypertension

**DOI:** 10.1186/s12931-021-01708-w

**Published:** 2021-05-04

**Authors:** Yanxia Wang, Xiaoming Li, Wen Niu, Jian Chen, Bo Zhang, Xiumin Zhang, Yingmei Wang, Shaokang Dang, Zhichao Li

**Affiliations:** 1 Department of Pathology, Xijing Hospital and School of Basic Medicine, Fourth Military Medical University, Xi’an, Shaanxi 710032 People’s Republic of China; 2 Department of Pathophysiology, School of Basic Medicine, Fourth Military Medical University, 169 Changle Western Street, Xi’an, Shaanxi 710032 People’s Republic of China; 3 Northwest University School of Medicine, Xi’an, Shaanxi 710075 People’s Republic of China; 4 Department of Respiratory and Critical Care, Tangdu Hospital, Fourth Military Medical University, 569 Xinsi Street, Xi’an, Shaanxi 710038 People’s Republic of China; 5 Department of Pathophysiology, Xi’an Peihua University, Xi’an, Shaanxi 710032 People’s Republic of China

**Keywords:** Alveolar epithelial cells, Hypoxic pulmonary hypertension, Pulmonary vascular remodeling and constriction, Reactive oxygen species

## Abstract

**Background:**

Hypoxic pulmonary hypertension (HPH) is a common type of pulmonary hypertension and characterized by pulmonary vascular remodeling and constriction. Alveolar epithelial cells (AECs) primarily sense alveolar hypoxia, but the role of AECs in HPH remains unclear. In this study, we explored whether AECs are involved in pulmonary vascular remodeling and constriction.

**Methods:**

In the constructed rat HPH model, hemodynamic and morphological characteristics were measured. By treating AECs with hypoxia, we further detected the levels of superoxide dismutase 2 (SOD2), catalase (CAT), reactive oxygen species (ROS) and hydrogen peroxide (H_2_O_2_), respectively. To detect the effects of AECs on pulmonary vascular remodeling and constriction, AECs and pulmonary artery smooth cells (PASMCs) were co-cultured under hypoxia, and PASMCs and isolated pulmonary artery (PA) were treated with AECs hypoxic culture medium. In addition, to explore the mechanism of AECs on pulmonary vascular remodeling and constriction, ROS inhibitor N-acetylcysteine (NAC) was used.

**Results:**

Hypoxia caused pulmonary vascular remodeling and increased pulmonary artery pressure, but had little effect on non-pulmonary vessels in vivo. Meanwhile, in vitro, hypoxia promoted the imbalance of SOD2 and CAT in AECs, leading to increased ROS and hydrogen peroxide (H_2_O_2_) production in the AECs culture medium. In addition, AECs caused the proliferation of co-cultured PASMCs under hypoxia, and the hypoxic culture medium of AECs enhanced the constriction of isolated PA. However, treatment with ROS inhibitor NAC effectively alleviated the above effects.

**Conclusion:**

The findings of present study demonstrated that AECs were involved in pulmonary vascular remodeling and constriction under hypoxia by paracrine H_2_O_2_ into the pulmonary vascular microenvironment.

## Background

Pulmonary hypertension is a progressive disorder and characterized by pulmonary vascular remodeling and vasoconstriction [1–3], which ultimately leads to right ventricular hypertrophy and right heart failure, as well as morbidity and mortality [4–6]. Hypoxic pulmonary hypertension (HPH) is highly prevalent in advanced chronic obstructive pulmonary disease and high altitude hypoxia [7], and has a complex pathological process involving multiple mechanisms. Although there are many studies on the mechanism of HPH, the underlying molecular mechanisms are not yet fully elucidated to this date. Therefore, it is highly needed to further explore novel mechanism of HPH.

It is well known that the pulmonary vascular system responds to hypoxia with vasoconstriction rather than vasodilation like in the systemic circulation [8]. Alveolar hypoxia causes the increase of pulmonary artery pressure, but there is no systemic circulation pressure increase. Alveolar hypoxia is primarily sensed at the alveolo capillary membrane, which is formed by juxtaposed epithelial and endothelial membranes in the alveolar wall, from where the hypoxic signal is propagated to pulmonary arterioles and subsequently triggers constriction of pulmonary artery smooth cells (PASMCs) [9]. In the lung, although alveolar epithelial cells (AECs) and endothelial cells were responsive to altered oxygen tensions, AECs were generally more sensitive than endothelial cells in regulation of hypoxic adaptation [10]. In the alveolar wall, the tight junction of epithelium and endothelium strongly indicates the presence of direct crosstalk [9]. Recently, the key role of endothelial cell-SMC crosstalk on HPH has been proposed [8, 11–15]. However, it is almost unknown whether AECs are involved in the development of HPH. The aim of the study was to analyze the role and possible mechanisms by which AECs are involved in HPH.

The electron transfer process produces reactive oxygen species (ROS) such as hydroxide radicals (HO^•^), singlet oxygen (^1^O2), superoxide anions (O_2_^**·**_^) and hydrogen peroxide (H_2_O_2_) [16–18]. In the cell normal metabolism, the antioxidant enzymes superoxide dismutase (SOD), catalase (CAT) and peroxidase (POD) can prevent the accumulation of O_2_^**·**_^ and H_2_O_2_ [19, 20]. The balance between production and degradation rates determines the steady-state concentrations of ROS in each intracellular compartment. H_2_O_2_ is a stable and easily diffused out of the cell [18]. H_2_O_2_ has been shown to be involved in cell proliferation [21], cell-cycle arrest [22] and apoptosis [23] and vascular smooth muscle constriction [24].The multiple responses of the lung to oxygen are a physiological paradigm of the variety of cellular responses to ROS in vitro. In the alveolus, AECs are the first to sense changes of oxygen concentration. Recently, there is evidence that hypoxia leads to a significant increase in the concentration of ROS in AECs [15, 25]. A recent study has shown that cell proliferation can be achieved only by exposing cells to a constant flow of H_2_O_2_ generated by the the glucose/glucose oxidase (G/GO) system [26]. Therefore, AECs, as the cells around the pulmonary capillaries, can affect the pulmonary vascular remodeling and constriction by constant release of H_2_O_2_.

In this study, we reported that AECs were involved in pulmonary vascular remodeling and constriction by paracrine H_2_O_2_ to pulmonary vascular microenvironment. And the upregulation of H_2_O_2_ in AECs was caused by imbalance of SOD2 and CAT under hypoxia. We hypothesized that the up-regulation of H_2_O_2_ in AECs could provide a novel mechanism into the pathogenesis of HPH.

## Methods

### Experimental protocol

Male Sprague–Dawley rats (200–250 g) were purchased from the animal center of the Fourth Military Medical University (Xi'an, China). All animal experimental procedures were approved by Animal Care and Use Committee of the Fourth Military Medical University and complied with the Declaration of the National Institutes of Health Guide for Care and Use of Laboratory Animals (certificate No. 201000082, Grade II). To obtain pulmonary hypertension rats, rats were housed intermittently in a hypobaric hypoxia chamber depressurized to 380 mmHg (10% oxygen) and subjected to hypobaric hypoxia challenge of 8 h/d for 4 weeks. Age-matched rats were housed in room air (21% oxygen) accordingly as controls.

Hemodynamic and histological analysis: the measurement of right ventricular pressure and the hematoxylin and eosin staining of lung and renal tissues were performed according to our established protocol [27]. Briefly, after 4 weeks of hypoxia, the animals were anesthetized with 20% ethylurethan (4 ml/ kg, i.p.). Then, a soft silica gel catheter linked to a Power lab system (AD Instruments, Colorado Springs, CO, Australia) was inserted into the right jugular vein. The right ventricle peak systolic pressure (RVSP) waveforms were showed on the monitor when the catheter arrived in the right ventricle chamber. Meanwhile, the mean carotid artery pressure (mCAP) was recorded via a special catheter inserted into the carotid artery. After the weight of right ventricle (RV) and left ventricle plus septum (LV + S) were obtained, the ratio of RV/ (LV + S) was calculated as an index of RV hypertrophy. The right lung and kidney were placed in neutral buffer (pH 7.4) containing 10% formalin for 72 h embedded in paraffin, sectioned into 5 µm thick sections and then subjected to hematoxylin and eosin staining. Microscopic evaluation showed structure remodeling of the pulmonary vessel. Total 60 of pulmonary vessel, bronchial vessel and renal vessel in approximate round shape were obtained from each group. Their external diameters are 50–180 µm and the average size of artery was 78 µm. The outside diameter and inside diameter of pulmonary vessel were measured by an image-processing program (Image-Pro Plus, Version 5.1, Media Cybernetics, Rockville, MD, USA). The medial wall thickness, the cross-sectional area of medial wall, and the total cross-sectional vessel area were obtained. Pulmonary vascular structure remodeling was assessed by percent medial wall thickness (MT %) and percent medial wall area (MA %) two indices: MT% = 100 × (2 × medial wall thickness) / (vessel diameter), MA% = 100 × (cross sectional medial wall area) / (total cross-sectional vessel area). All the morphological analysis was conducted via a double-blind method.

Arterial rings isolation: the PA and aortic artery (AA) were obtained according to published articles [25]. Briefly, normoxic and HPH rats were anesthetized with 20% ethylurethan (4 ml/kg, i.p.). Lung and heart were removed and immersed into ice-cold oxygenated Krebs–Henseleit solution (in mM: NaCl 127, KCl 4.7, NaHCO_3_ 17, MgSO_4_ 1.17, KH_2_PO_4_ 1.18, CaCl_2_ 2.5, d-glucose 5.5) after median sternotomy was performed. Under a dissecting microscope, the third-division (external diameter < 300 μm) PA and AA were isolated carefully, cleared of connective tissue and cut into 3-mm-length rings. Endothelium was removed by gently rubbing the lumen with swab in rings. The PA and AA rings were suspended respectively on stainless steel hook connected to force transducers (AD Instruments, Colorado Springs, CO) for isometric force recording in individual water jacketed organ chamber containing modified Krebs–Henseleit solutions parged continuously with 95% O_2_ and 5% CO_2_ at 37 °C. Isometric force was recorded with a force–displacement transducer and a PowerLab eight-channel data acquisition system (AD Instruments, Colorado Springs, CO, USA) and then was analyzed by MacLab/400 and Chart software (version 5.5 from ADInstruments).The pulmonary and aortic artery ring were stretched to a predetermined optimal passive tension of 500 mg and 1000 mg, respectively and stabilized for 60 min with three washouts at 20-min intervals. Then reproducibility of contractile responses to 1 µM phenylephrine (> 300 mg) was established. The rings were rinsed with Krebs–Henseleit solution and the tension returned to baseline. To test the effects of H_2_O_2_ on the constriction of PA and AA, dose–response curve was obtained by cumulative addition of exogenous H_2_O_2_ (5 × 10^–6^ to 10^–3^ M) at 10-min interval on PA and AA rings. The H_2_O_2_ concentration of the largest contractile force of artery was selected and then treated with ROS inhibitor N-acetylcysteine (NAC) for 10 min to observe the change of contractile force. To further confirm that AECs participated in the constriction of PA and AA, pulmonary and aortic ring from normoxic and HPH rats were treated with AECs normoxic and hypoxic culture medium and NAC at 10-min intervals to observe the change of contractile force. The medium with 5% fetal bovine serum (FBS) was used as a negative control. The experiments involving NAC and H_2_O_2_ were performed in the dark. For the vasoconstriction experiment, 1 μmol/L PE was used. Vascular ring contraction to maximum was labeled as 100%, and the contraction data for each group was expressed as a percentage of the maximum contraction caused by 1 μM PE.

### Cell culture

Rat primary PASMCs and AASMCs were obtained by tissue explants culturing method according to our established protocol [22]. PA and AA were isolated from male Sprague–Dawley rats (200–250 g) and dissected into small pieces after the adventitial layers were removed. Then, they were placed in an overturned culture flask containing Dulbecco Eagle’s minimum essential Medium (DMEM, HyClone, Logan, UT) with 15% FBS, CellMax, Beijing, China) at 37 °C in 21% oxygen condition. The flask was carefully turned over after 4 h. PASMCs and AASMCs crawled out from the tissue in about a week. Cells were used between passages 3 to 6. Smooth muscle cell was identified by positive staining for smooth muscle a-actin (mouse monoclonal antibody, Sigma-Aldrich, St. Louis, MO, USA) at each passage.

Alveolar epithelial cells (AECs) in this study were rat primary ATII cells. Rat primary ATII cells were isolated from male SpragueDawley rats (180–200 g) as previously described [28]. Pooled cells from 2 rats were prepared as follows. Rats were injected intraperitoneally with 20% ethylurethan (4 ml/ kg, i.p.) and intravenously 4000U/kg heparin sodium. Then, rats were exsanguinated by cutting the abdominal aorta under the aseptic condition. Blood was replaced by solution II (50 ml) (in mM: 140 NaCl, 5 KCl, 10 Hepes, 2CaCl_2_, 2.5 PBS (pH 7.4), 1.3 MgSO_4_) which was perfused via the PA. The lungs were removed from the thorax and lavaged and washed 8 times with the solution I (10 ml/time) (in mM: 140NaCl, 5 KCl, 10 Hepes, 6 D-glucose, 2.5 PBS (pH 7.4), 0.2 EDTA) and 2 times with the solution II (10 ml/time). Lungs were then filled 2 times with trypsin solution (10 ml/time) prepared in solution II and incubated in incubator for 10 min at 37℃ every time. The lungs were dissected into small pieces in the presence of 4 ml DNAse I and 5 ml FBS. The lung tissues were then sequentially filtered through 125 μm and 75 μm stainless mesh. The filtrate was centrifuged at 1000r/min for 8 min. The cell pellet was resuspended in DMEM at 37℃.The cell suspension was plated at a density of 2 × 10^6^ cells/ml in 25 cm^2^ bacteriologic plastic dishes coated rat IgG to remove macrophages and incubated at 37℃ in 5% CO_2_ incubator for 1 h. The unattached cells in suspension were removed and centrifuged at 1000 r/min for 8 min. The cell pellet was plated at a density of 5 × 10^5^ cells/cm^2^ in 6-well culture plates with DMEM, 15% FBS, 100 U/ml penicillin and 100 U/ml streptomycin and incubated at 37℃ under 5% CO_2_ incubator. The cell purity after 24 h was assessed by a characteristic fluorescence with phosphine3 Ras previously described [28]. AECs were seeded in 24-well culture plates at 5 × 10^3^ cells per well under normoxic (21% O_2_) or hypoxic conditions (5% O_2_). AECs culture medium was collected at 24 h for the subsequent experiments.

### Cell treatment

The effect of hypoxia on the proliferation of PASMCs and AASMCs was investigated under different oxygen concentrations. PASMCs and AASMCs were seeded in 96-well culture plates at 3 × 10^4^ cells per well under normoxic (21% O_2_) or hypoxic conditions (10% O_2_ and 5% O_2_).

The effect of AECs on the proliferation of PASMCs and AASMCs was investigated using 24-well transwell insert co-culture model (0.4 μm pore). PASMCs and AASMCs were seeded in 24-well culture plates at a density of 3 × 10^4^ cells per well, ATII were seeded in transwell inserts at a density of 1 × 10^4^ cells per insert. The cells were maintained under normoxia (21% O_2_) or hypoxia conditions (5% O_2_). The cells were grouped as follows: (1) normoxia (2) hypoxia (3) co-culture with ATII under normoxia (4) co-culture with ATII under hypoxia.

To further confirm that AECs participated in the proliferation of PASMCs and AASMCs, the culture medium of PASMCs and AASMCs were replaced by AECs culture medium under normoxic or hypoxic conditions every 12 h. The cells were grouped as follows: (1) normoxia (2) hypoxia (3) treatment with ATII normoxic culture medium under normoxia (4) treatment with ATII hypoxic culture medium under normoxia (5) treatment with ATII normoxic culture medium under hypoxia (6) treatment with ATII hypoxic culture medium under hypoxia.

We assessed the effects of H_2_O_2_ on the proliferation of PASMCs and AASMCs by the cumulative addition of exogenous H_2_O_2_ (5 × 10^–6^ to 10^–3^ M).The H_2_O_2_ concentration of the largest proliferation of PASMCs and AASMCs was selected and then treated with NAC (5 mMand10mM) to observe the change of proliferation. As well as NAC (10 mM) was used in the co-culture system.

To better investigate the effects of hypoxia on cell proliferation, direct cell counting was performed. Cells were seeded 6 × 10^4^ cells in 6-well plates and cultured overnight. The cells were then incubated in serum-free medium for 24 h. Cells were cultured with normoxic (21% O_2_) or hypoxic conditions (10% O_2_ and 5% O_2_), and after 48 h, they were were rinsed with phosphate buffered solution, harvested by mild trypsinization, and counted with a hematocytometer.

### MTT assay

All cells were starved in serum-free medium for 24 h after growing to subconfluence and then cultured with 5% fetal bovine serum for 48 h under normoxic or hypoxic conditions. Subsequently, MTT (5 mg/mL) was added into the plates (80 μL/well in 24-well plates or 20 μL/well in 96-well plates) and incubated for another 4 h. Dimethyl sulfoxide was added into each well, and all plates were shaken for 10 min in a shaker. The optical density (OD) values were collected using a spectrophotometer (PowerWave XS, BioTekInc, Winooski, VT).

### Quantitative real-time PCR (qRT-PCR)

qRT-PCR was performed with SYBR PrimeScript™ RT-PCR kit (TakaRa, Dalian, China). The total RNA of cells was extracted using Trizol (Invitrogen, Carlsbad, CA, USA). First-strand cDNA was synthesized from RNA. GAPDH was used as an internal control. Primer sequences were designed using the Primer Premier 5.0 software (PREMIER Biosoft International, CA, USA) and synthesized by the DNA Bio Tec (Shanghai, China). Primers sequences used were asfollows: forward: 5′-TGGGAAACAACACCCCTATTTT-3′ and reverse: 5′-CGAAGATACCACCAGTCGTAGTTG-3′ for CAT; forward: CTGTGGCTGAGCTGTTGTAA and reverse: ACAGCGTCCAAGCAATTCAA for SOD_2_; forward: 5′-CTATCGGCAATGAG CGGTTC-3′and reverse: 5′–GATCTTGATCTTCATGGTGCTAGG-3′for GAPDH.

### ROS, H_2_O_2_ and SOD measurement

PASMCs and AASMCs were stained with an oxidant-sensitive fluorescence dye DCFH-DA (10 μmol/L, Nanjing Jian Cheng Bioengineering Institute, Nanjing, China). Subsequently, the intracellular total ROS were detected through fluorescence microscopy (Leica, Heidelberg, Germany) and flow cytometry.

The content of H_2_O_2_ in AECs and AECs culture medium were detected using a commercially available Hydrogen Peroxide Assay Kit (Beyotime Inc, Jiangsu, China) according to the recommended protocols. The concentrations of H_2_O_2_ in different groups were finally normalized to the corresponding protein concentrations.

The content and activity of SOD in AECs were detected using a commercially Tatal Superoxide Dismutase Assay Kit with WST-8 (Beyotime Inc, Jiangsu, China) according to the recommended protocols. The content of SOD in different groups was finally normalized to the corresponding protein concentrations. The activity of SOD is calculated by the formula.

### Statistical analysis

All data were analyzed using SPSS 20.0 software, and represented as average ± standard deviation (mean ± SD). Analysis of variance (one-way ANOVA) was used for multiple group comparisons, and paired t test was used for two group comparisons. A *P* value < 0.05 was considered statistically significant.

## Results

### Effects of hypoxia on hemodynamics and pulmonary and systemic arterial wall remodeling

**Fig. 1:**
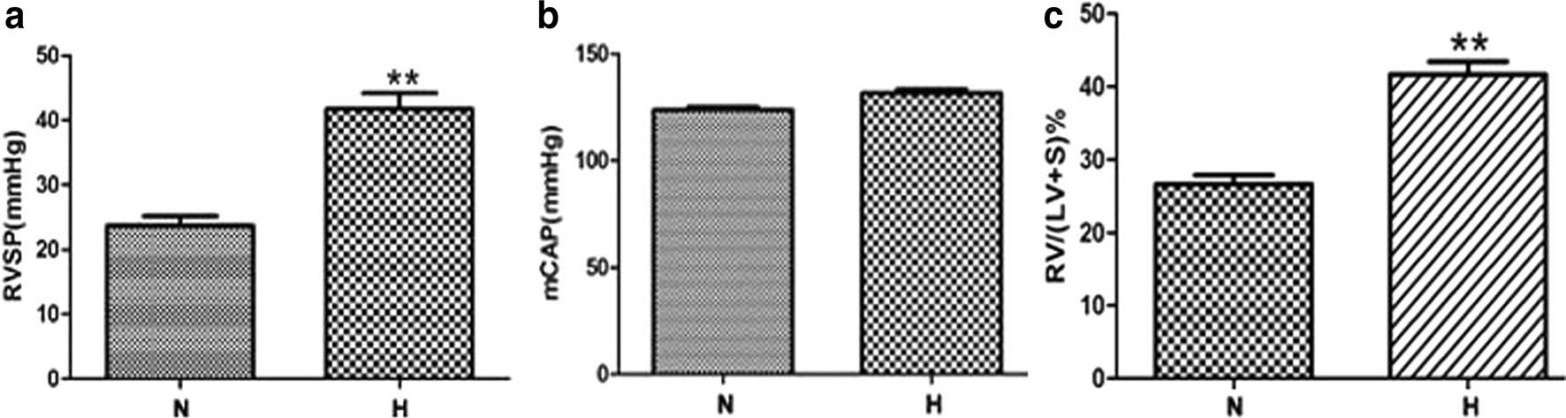
Effects of hypoxia on RVSP, mCAP and right ventricular hypertrophy index in rats. **a** RVSP increased significantly in hypoxic group compared with the normoxic group. **b** Hypoxia had no significant effect on mCAP. **c** RV/(LV + S)% increased significantly in hypoxic group compared with normoxic group. N:  normoxic group, H:  hypoxic group. n = 12, Data are means ± S.D. **P < 0.01 vs. N

**Fig. 2:**
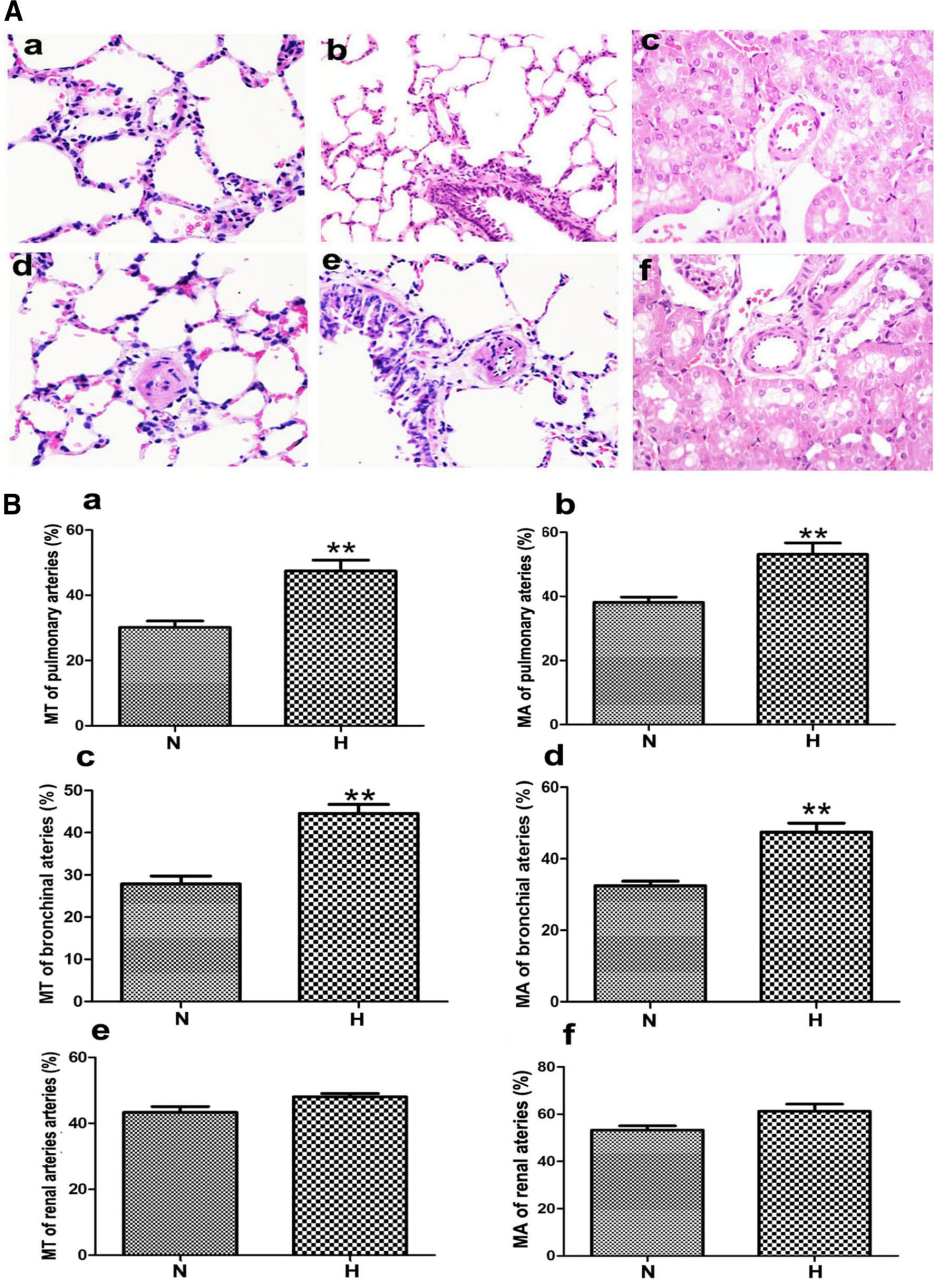
Effects of hypoxia on structure of pulmonary and systemic vessels in rats. **A** Reconstruction of pulmonary and systemic artery were observed by HE staining (**a**–**c**) pulmonary artery, bronchial artery and renal artery of normoxic group (**d**–**f**) pulmonary artery, bronchial artery and renal artery of hypoxic group. **B** MT% and MA% of pulmonary and bronchial artery were significantly increased in hypoxic group compared with the normoxic group (**a**, **b**) MT% and MA% of pulmonary artery (**c**, **d**) MT% and MA% of bronchial artery (**e**, **f**) MT% and MA% of renal artery. N:  normoxic group, H: hypoxic group. n = 12, Data are means ± S.D. **P < 0.01 vs*.* N

**Fig. 3:**
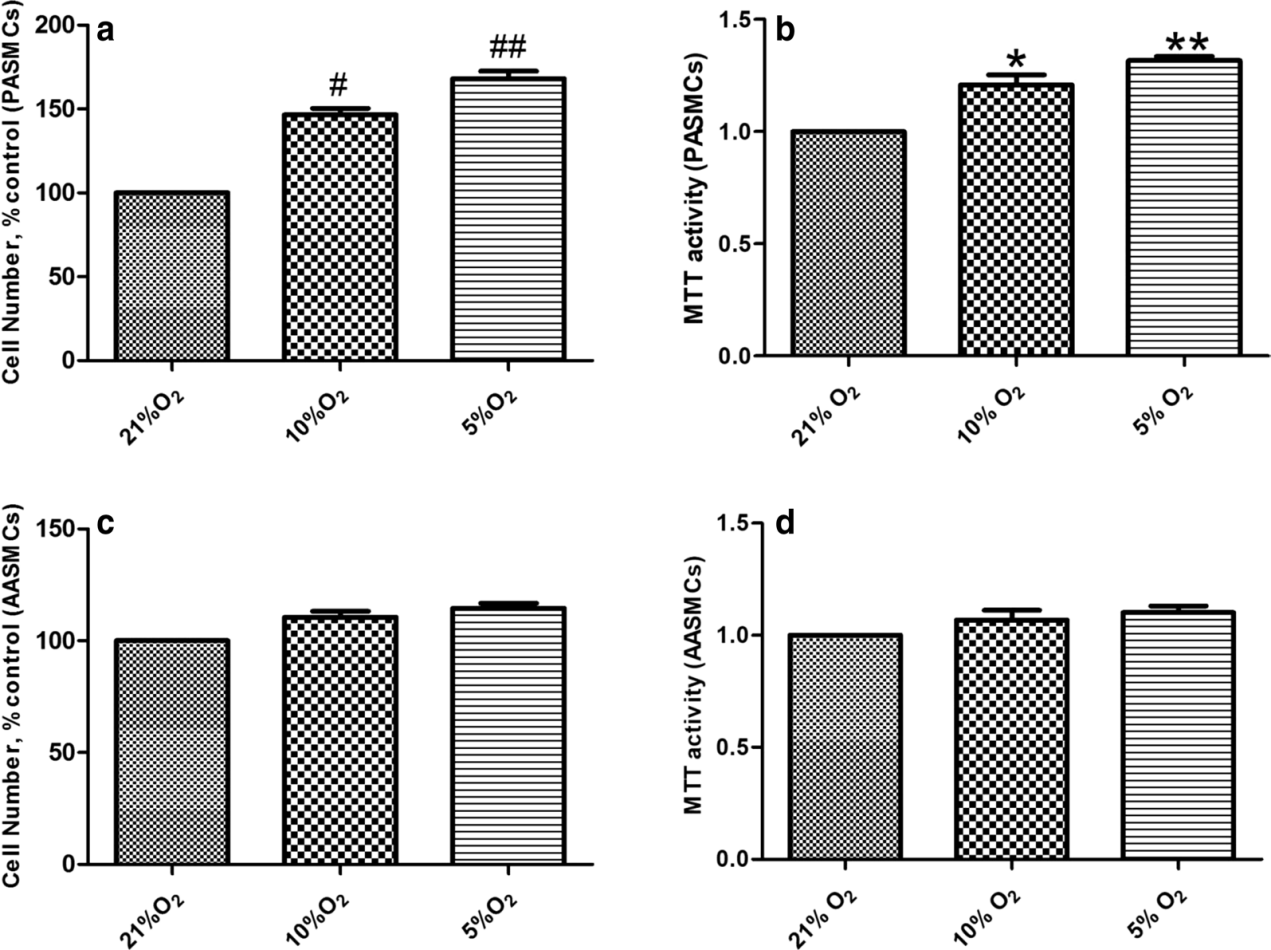
Effects of hypoxia on proliferation of PASMCs and AASMCs. **a** and **b** Hypoxia promoted the proliferation of PASMCs, **c** and **d** Hypoxia had no significant effect on the proliferation of AASMCs. Direct cell counting was performed in **a** and **c**, MTT assay was performed in **b** and **d**. The growth of PASMCs or AASMCs under 21% O_2_ (control) was set as 100% and the proliferation of treated cells was calculated as a percentage of them. n = 8, Data are means ± S.D. ^#^P < 0.05 or ^##^P < 0.01 vs. 21% O_2_,*P < 0.05 or **P < 0.01 vs. 21% O_2_

RVSP was measured by catheterization via jugular vein to right ventricle, which substitutes for the pulmonary artery pressure. Compared with normoxic group, the RVSP (Fig. 1a) and RV/ (LV + S) % (Fig. 1c) increased significantly in the hypoxia group, while the effect of hypoxia on mCAP was not significant (Fig. 1b). Compared with the normoxia group, the MT% and MA% of PA and BA in hypoxic group were significantly increased (Fig. 2a, b). However, hypoxia had no significant effect on the MT% and MA% of RA (Fig. 2a, b). Besides, compared with the normoxic group, hypoxia promoted the proliferation of PASMCs (Fig. 3a, b) in vitro, but did not affect the proliferation of AASMCs (Fig. 3c, d). These results indicated that the different responses of pulmonary and systemic arteries to hypoxia might be related to the vascular microenvironment.

### Effects of AECs on the proliferation of PASMCs and AASMCs under hypoxia

**Fig. 4:**
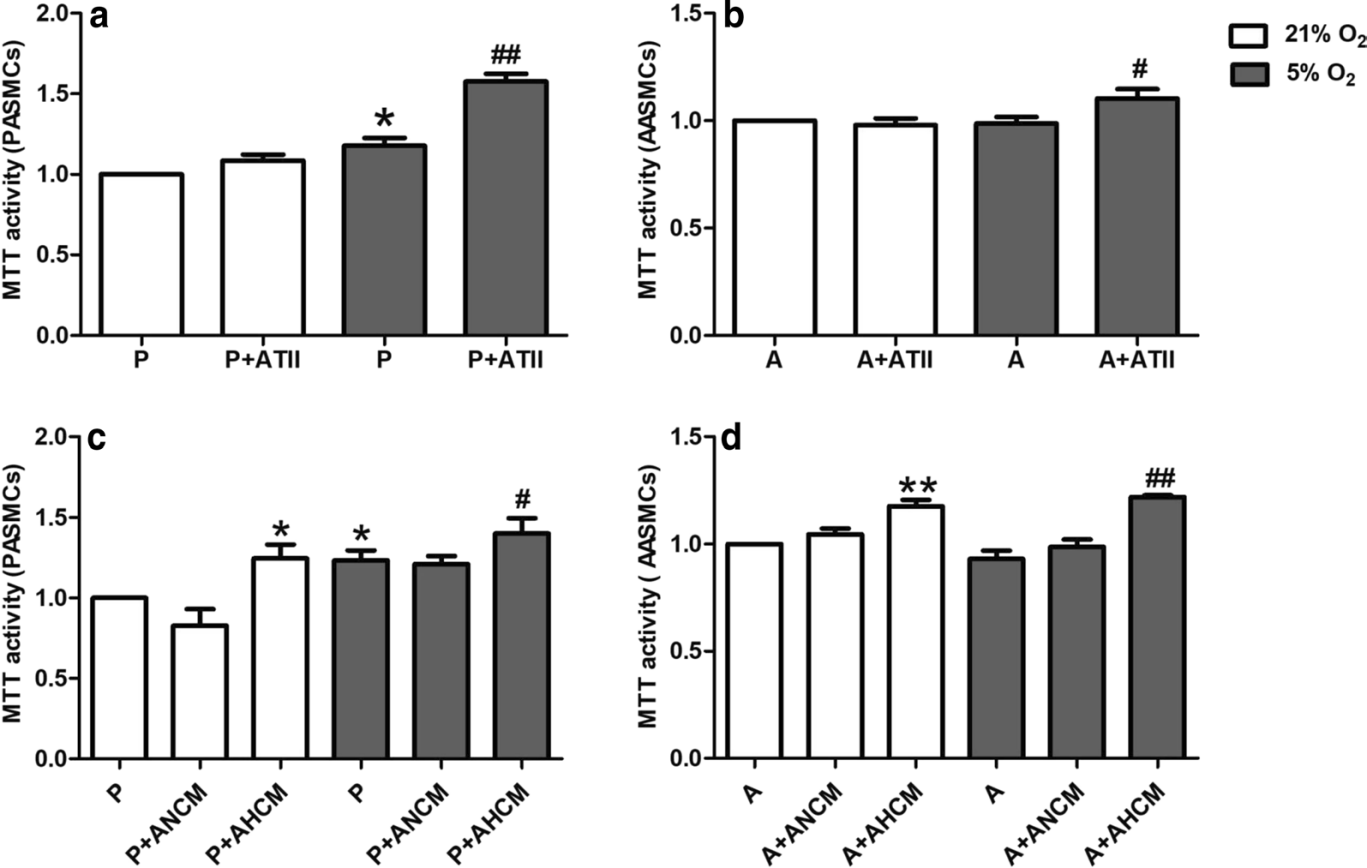
Effects of AECs on the proliferation of PASMCs and AASMCs under hypoxia. **a** Co-culture with ATII significantly promoted the proliferation of PASMCs under hypoxia. *P < 0.05 vs. P (21% O_2_), ^##^P < 0.01 vs. P (5% O_2_). **b** Co-culture with ATII significantly promoted the proliferation of AASMCs under hypoxia. ^#^P < 0.05 vs. A (5% O_2_). **c** ATII hypoxic culture medium significantly promoted the proliferation of PASMCs. *P < 0.05 vs. P (21% O_2_), ^#^P < 0.05 vs. P (5% O_2_) **d** ATII hypoxic culture medium significantly promoted the proliferation of AASMCs. **P < 0.01 vs. A (21% O_2_), ^##^P < 0.01 vs. A (5% O_2_). The growth of PASMCs or AASMCs under 21% O_2_ was set as 100% and the proliferation of treated cells was calculated as a percentage of them. P:  PASMCs, A:  AASMCs, *ANCM* ATII normoxic culture medium, *AHCM* ATII hypoxic culture medium, n = 8, Data are means ± S.D

To explore the effect of AECs on PASMCs or AASMCs in vitro, the proliferation of PASMCs and AASMCs were detected by MTT. Under normoxia, co-culture with ATII had no significant effect on the proliferation of PASMCs and AASMCs compared with those co-cultured without ATII (Fig. 4a, b), However, co-culture with ATII significantly promoted the proliferation of PASMCs and AASMCs compared with those co-cultured without ATII under hypoxia (Fig. 4a, b). To further clarify the effect of AECs on the proliferation of PASMCs or AASMCs, the culture medium of PASMCs and AASMCs were replaced with AECs conditioned culture medium. Under normoxia, the normoxic culture medium of ATII had no significant effect on the proliferation of PASMCs and AASMCs compared with those co-cultured without ATII culture medium (Fig. 4c, d). However, ATII hypoxic culture medium significantly promoted the proliferation of PASMCs and AASMCs compared with those co-cultured without ATII culture medium (Fig. 4c, d). Under hypoxia, the normoxic culture medium of ATII had no significant effect on the proliferation of PASMCs and AASMCs compared with those co-cultured without ATII culture medium (Fig. 4c, d). However, ATII hypoxic culture medium significantly promoted the proliferation of PASMCs and AASMCs compared with those co-cultured without ATII culture medium (Fig. 4c, d). These data indicated that AECs could promote the proliferation of PASMCs and AASMCs in hypoxia.

### Effects of AECs conditioned culture medium on the constriction of PA and AA rings

**Fig. 5:**
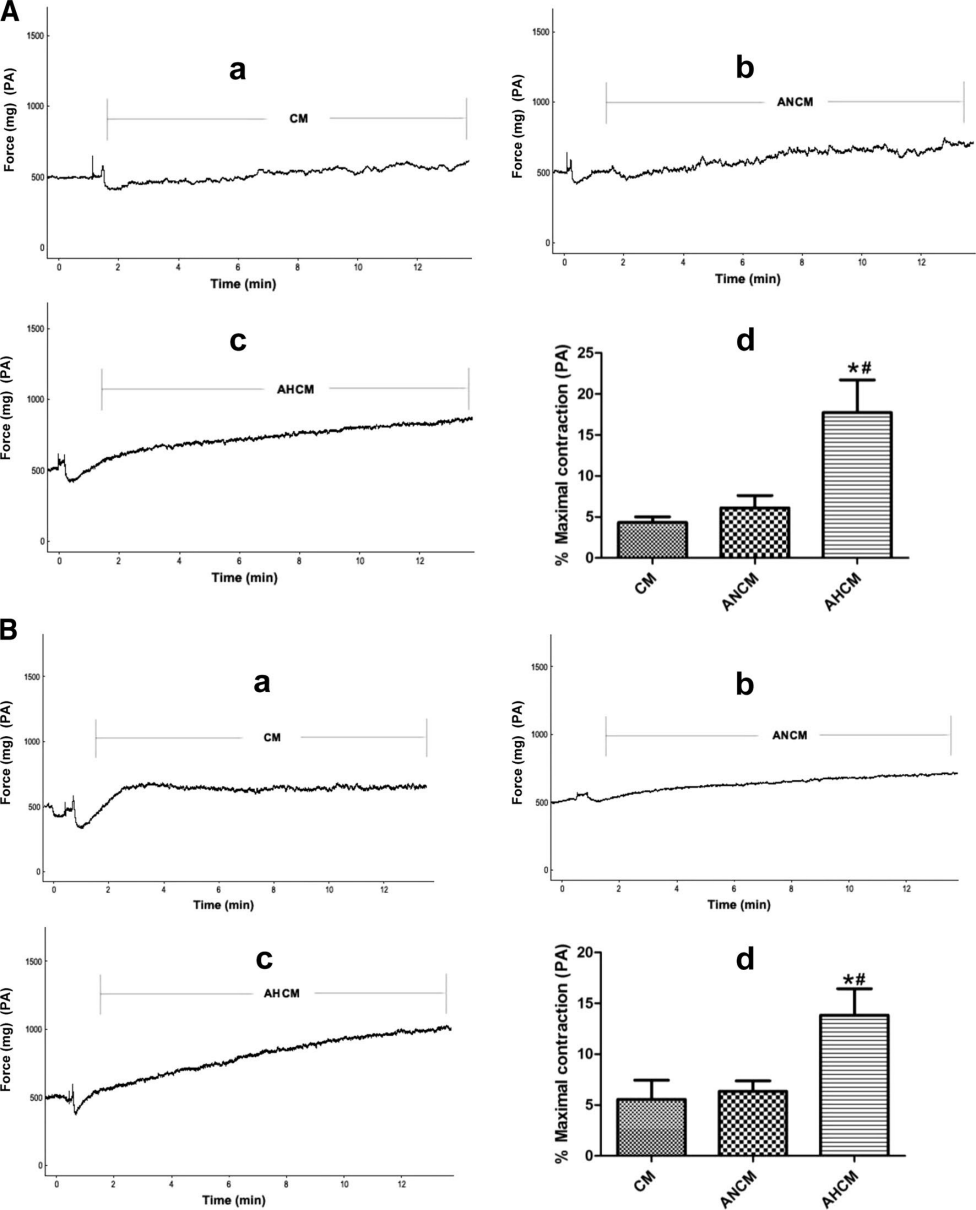
Effects of AECs conditioned culture medium on the constriction of PA ring. **A** ATII hypoxic hypoxic culture medium significantly promoted the constriction of PA ring from normoxic rats (**a**–**c**) the representative curve for the effect of culture medium, ATII normoxic and hypoxic culture medium on the constriction of PA ring from normoxic rats (**d**) the summarized data of PA ring maximum constriction. **B** ATII hypoxic culture medium significantly promoted the constriction of PA ring from HPH rats (**a**–**c**) the representative curve for the effect of culture medium, ATII normoxic and hypoxic culture medium on the constriction of PA ring from HPH rats (**d**) the summarized data of PA ring maximum constriction. *CM*  culture medium, *ANCM*  ATII normoxic culture medium, *AHCM*  ATII hypoxic culture medium, n = 5, Data are means ± S.D. *P < 0.05 vs. CM, ^#^P < 0.05 vs. ANCM

**Fig. 6:**
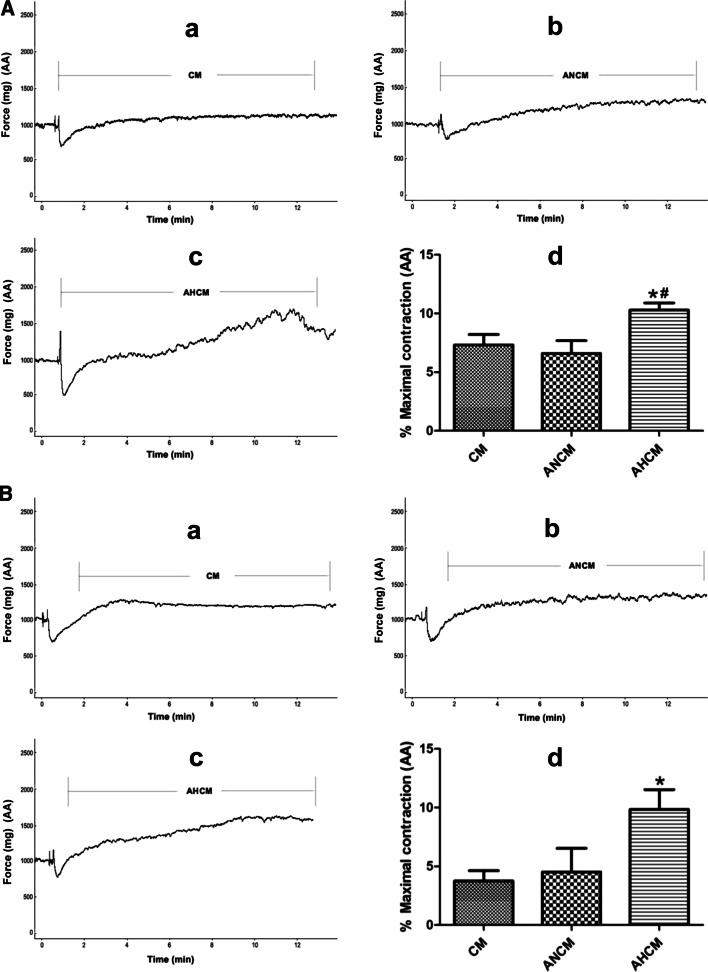
Effects of AECs conditioned culture medium on the constriction of AA ring. **A** ATII hypoxic culture medium significantly promoted the constriction of AA ring from normoxic rats, (**a**–**c**) the representative curve for the effect of culture medium, ATII normoxic and hypoxic culture medium on the constriction of AA ring from normoxic rats, (**d**) the summarized data of AA ring maximum constriction. **B** ATII hypoxic culture medium significantly promoted the constriction of AA ring from HPH rats, (**a**–**c**) the representative curve for the effect of culture medium, ATII normoxic and hypoxic culture medium on the constriction of AA ring from HPH rats, (**d**) the summarized data of AA ring maximum constriction. *CM* culture medium, *ANCM*  ATII normoxic culture medium, *AHCM*  ATII hypoxic culture medium, n = 5, Data are means ± S.D.*P < 0.05 vs. CM, ^#^P < 0.05 vs. ANCM

To explore whether AECs culture medium could regulate PA and AA rings, we further examined the effects of AECs culture medium on PA or AA rings from normoxic and HPH rats. For normoxic rats, the normoxic culture medium from ATII had no significant effect on the constriction of PA (Fig. 5a) and AA rings (Fig. 6a) compared with the culture medium group. However, the hypoxic culture medium from ATII significantly promoted the constriction of PA (Fig. 5a) and AA rings (Fig. 6a) compared with the culture medium or the normoxic culture medium group. For HPH rats, the normoxic culture medium from ATII had no significant effect on the constriction of PA (Fig. 5b) and AA rings (Fig. 6b) compared with the culture medium group. However, the hypoxic culture medium from ATII significantly promoted the constriction of PA (Fig. 5b) and AA rings (Fig. 6b) compared with the culture medium or the normoxic culture medium group. These results indicated that AECs hypoxic culture medium could induce the constriction of PA and AA.

### Effects of hypoxia on ROS in AECs

**Fig. 7:**
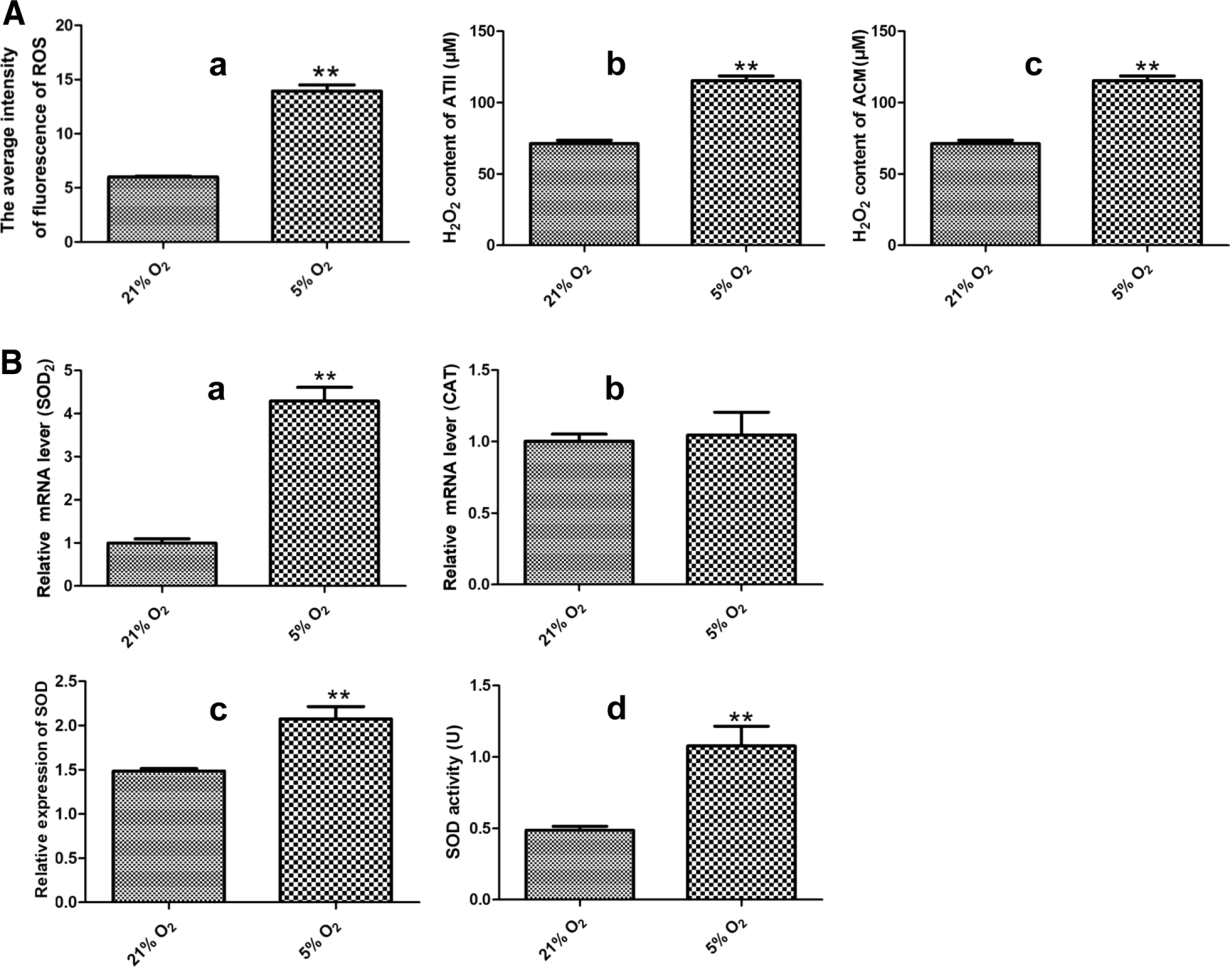
Effect of hypoxia on ROS in AECs. **A** Hypoxia significantly increased the intensity of ROS (**a**) and the content of H_2_O_2_ (**b**) in ATII and the content of H_2_O_2_ in ATII culture medium (**c**). (**B**) Hypoxia significantly increased *SOD2* mRNA level (**a**), the total content of SOD (**c**) and the activity of SOD (**d**) of ATII, but has no significant effect on the *CAT* mRNA level (**b**). n = 3, Data are means ± S.D. *ACM*  ATII culture medium **P < 0.01 vs. 21% O_2_

To explore the possible mechanism involved the effect of AECs on the proliferation of PASMCs and AASMCs and the constriction of PA and AA, total intracellular ROS was detected through DCFH-DA, and H_2_O_2_ in AECs and the culture medium were determined by commercial kit. Hypoxia significantly increased the production of ROS and H_2_O_2_ in AECs and AECs culture medium (Fig. 7a). Meanwhile, we examined mRNA level of *SOD2* and *CAT*, and detected the total level and activity of SOD in AECs by commercial kits. Hypoxia significantly increased the *SOD2* mRNA of (Fig. 7b) and the total level and activity of SOD (Fig. 7b). However, hypoxia has no significant effect on the *CAT* mRNA (Fig. 7b). These results indicated that the increase of ROS and H_2_O_2_ in AECs may be caused by imbalance of SOD2 and CAT in hypoxia.

### Effects of exogenous H_2_O_2_ and NAC intervention on the proliferation of PASMCs and AASMCs and the constriction of PA and AA rings

**Fig. 8:**
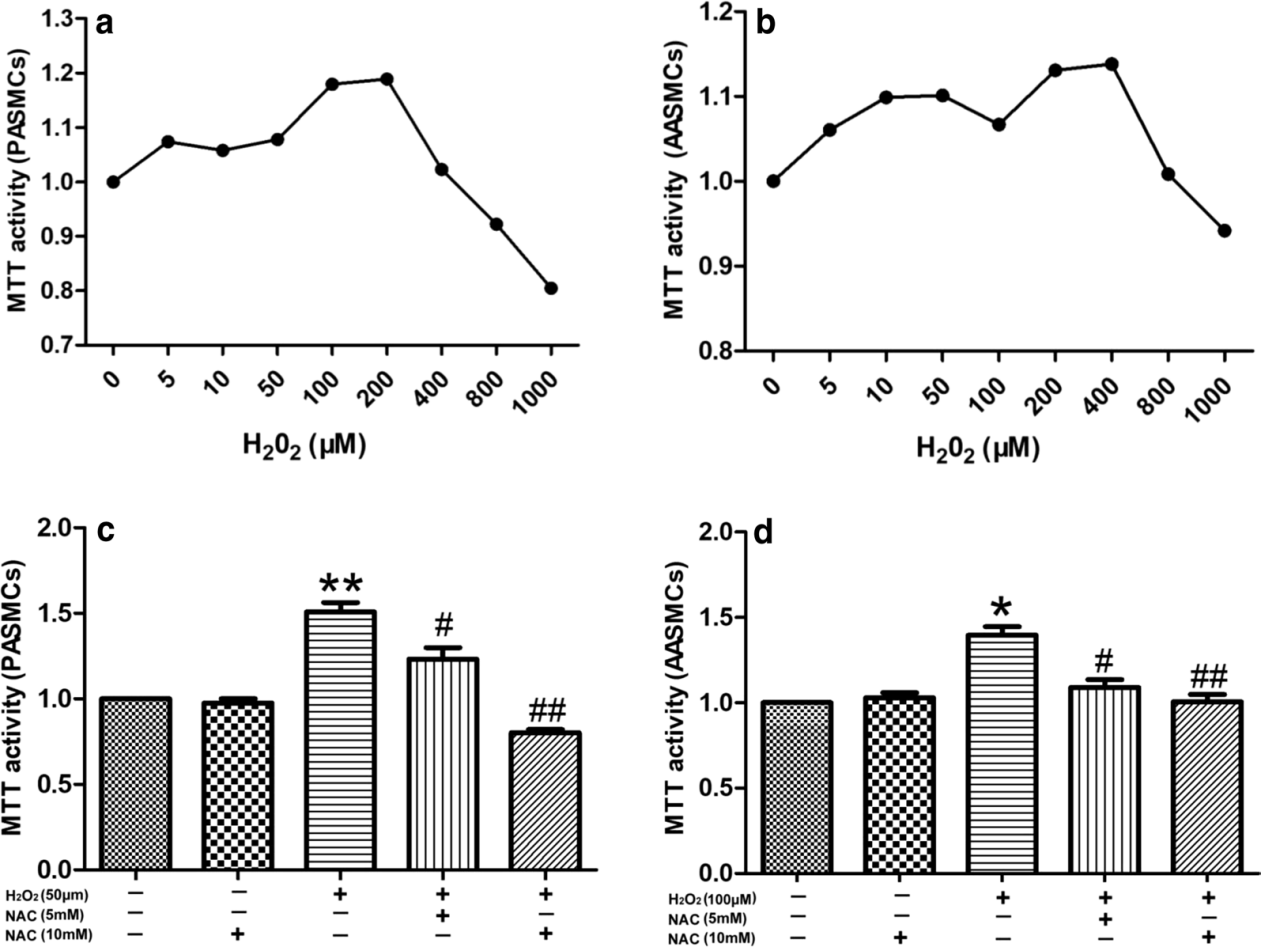
Effects of exogenous H_2_O_2_ and NAC intervention on the proliferation of PASMCs and AASMCs. **a** The effects of exogenous H_2_O_2_ on the proliferation of PASMCs was biphasic, the proliferation of PASMCs increased in a dose-dependent manner by 0 ~ 50 μM H_2_O_2_ and was gradually inhibited by 100 ~ 1000 μM H_2_O_2_. **b** The effects of exogenous H_2_O_2_ on the proliferation of AASMCs was biphasic, the proliferation of AASMCs increased in a dose-dependent manner by 0 ~ 100 μM H_2_O_2_ and was gradually inhibited by 200 ~ 1000 μM H_2_O_2_. **a** and **b **The cell growth of 0 μM H_2_O_2_ group was set as 100% and the proliferation of treated cells was calculated as a percentage of them. n = 8, Data are means ± S.D. **P < 0.01 or *P < 0.05 vs. 0 μM H_2_O_2_. **c** 5 mM and 10 mM NAC effectively inhibited the proliferation of PASMCs induced by 50 μM H_2_O_2_. **P < 0.01 vs. without any treatment group, ^##^P < 0.01 or ^#^P < 0.05 vs. treatment with 50 μM H_2_O_2_ alone group. **d** 5 mM and 10 mM NAC effectively inhibited the proliferation of AASMCs induced by 100 μM H_2_O_2_. *P < 0.05 vs. without any treatment group, ^##^P < 0.01 or ^#^P < 0.05 vs. treatment with 100 μM H_2_O_2_ alone group. (**c** and **d**) The cell growth of the group without any treatment was set as 100% and the proliferation of treated cells was calculated as a percentage of them. n = 8, Data are means ± S

Some studies have reported that the effects of different concentrations of H_2_O_2_ on cell proliferation were different. We used exogenous 0–1000 μM H_2_O_2_ to observe how H_2_O_2_ affects the proliferation of PASMCs andAASMCs under normoxia. The results showed that 0–50 μM H_2_O_2_ increased the proliferation of PASMCs in a dose-dependent manner. However, the proliferation of PASMCs was then gradually inhibited by 100–1000 μM H_2_O_2_ (Fig. 8a). Similarly, H_2_O_2_ (0–100 μM) also led to a dose-dependent increase in the proliferation of AASMCs, while H_2_O_2_ (200–1000 μM) inhibited the proliferation of AASMCs (Fig. 8b). To test the effect of NAC on PASMCs or AASMCs proliferation, we chose 50 μM and 100 μM H_2_O_2_, which mostly promoted the proliferation of PASMCs and AASMCs, respectively. The results showed that NAC (5 mM and 10 mM) effectively inhibited the proliferation of PASMCs and AASMCs, and 10 mM NAC was more effective (Fig. 8c, d).

**Fig. 9:**
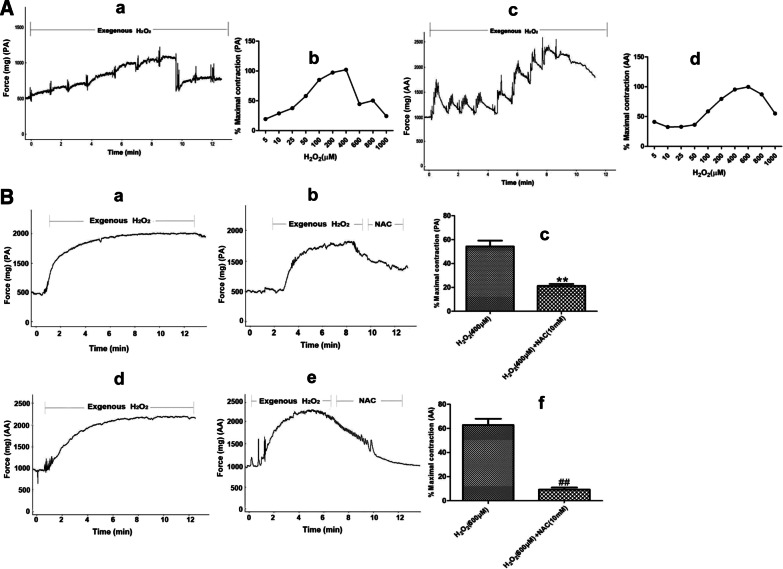
Effects of exogenous H_2_O_2_ and NAC intervention on the constriction of PA and AA rings. **A** The effects of exogenous H_2_O_2_ on the constriction of PA and AA rings was biphasic (**a**, **c**) the representative curve for the effect of 5 ~ 1000 μM exogenous H_2_O_2_ on the the constriction of PA and AA rings (**b**, **d**) the summarized data of PA and AA rings maximum constriction, the constriction of PA ring increased in a dose-dependent manner by 5 ~ 400 μM H_2_O_2_ and was gradually inhibited by 600 ~ 1000 μM H_2_O_2_, the constriction of AA ring increased in a dose-dependent manner by 5 ~ 600 μM H_2_O_2_ and was gradually inhibited by 800 ~ 1000 μM H_2_O_2_. **B** 10 mM NAC effectively inhibited the constriction of PA and AA rings (**a**, **b**, **d**, **e**) the representative curve for the effect of H_2_O_2_ and NAC intervention on the constriction of PA and AA rings (c, f) the summarized data of PA and AA rings maximum vasodilation, n = 5, Data are means ± S.D. **P < 0.01 vs. 400 μM H_2_O_2_, ^##^P < 0.01 vs. 600 μM H_2_O_2_

Exogenous H_2_O_2_ (5–1000 μM) was used to explore whether H_2_O_2_ affects the constriction of PA and AA rings in normal rats. The results showed that 5–400 μM H_2_O_2_ increased the constriction of PA ring in a dose-dependent manner, while the constriction of PA ring was gradually inhibited by 600–1000 μM H_2_O_2_ (Fig. 9a). Similarly, H_2_O_2_ (5–600 μM) also increased the constriction of AA rings in dose-dependent manner, and then inhibited the constriction of AA ring at 800–1000 μM (Fig. 9a). To test the effect of NAC on PA and AA rings constriction, we then chose 400 μΜ and 600 μM H_2_O_2_, both of which mostly affected the constriction of PA (Fig. 9b) and AA rings (Fig. 9b), respectively. The results showed that 10 mM NAC effectively inhibited the constriction of PA and AA (Fig. 9b).

### Effects of NAC intervention on the proliferation of PASMCs and AASMCs and the constriction of PA and AA rings

**Fig. 10:**
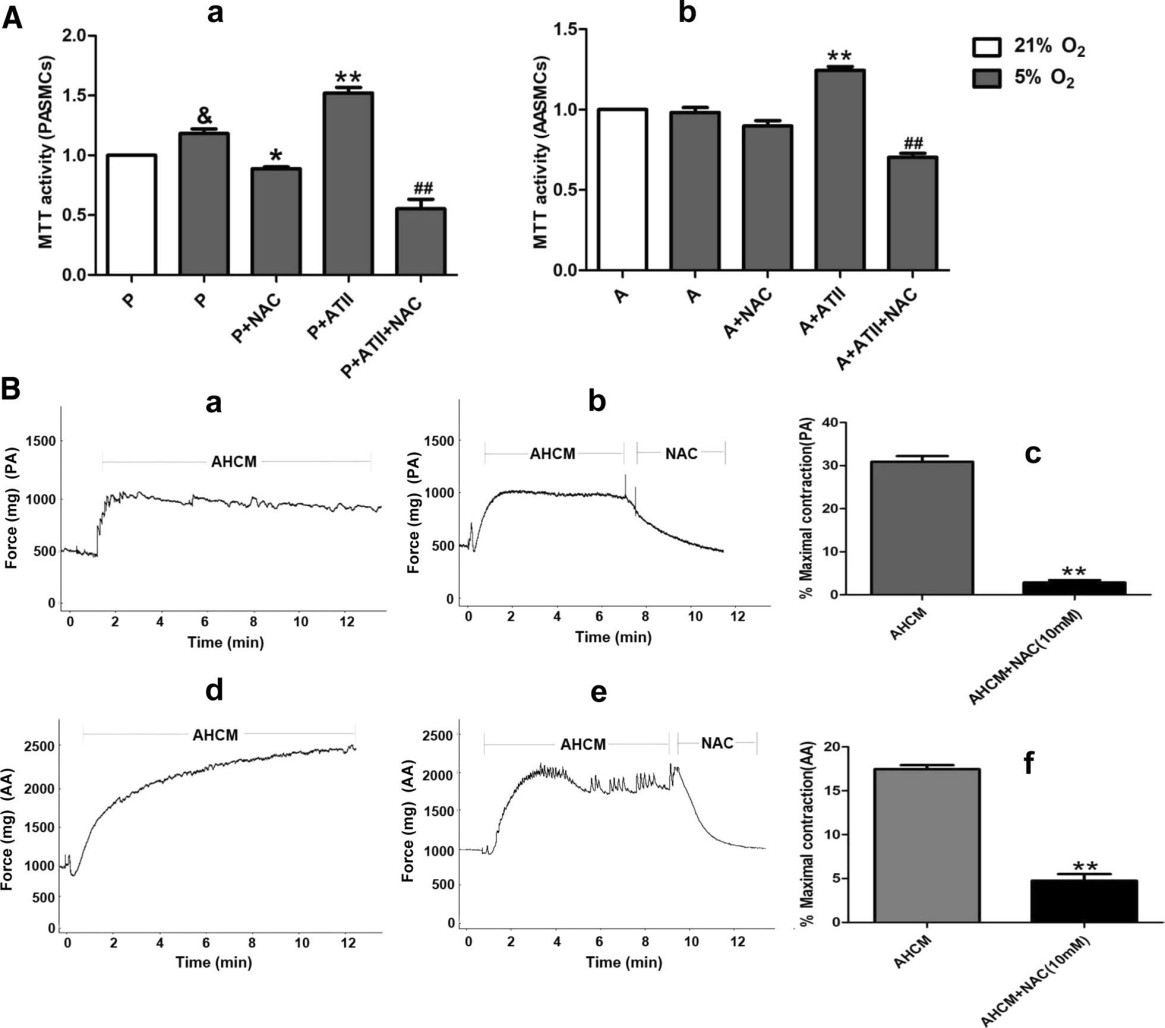
Effects of NAC intervention on the proliferation of PASMCs and AASMCs and the constriction of PA and AA rings. **A** NAC inhibited the proliferation of PASMCs (**a**) and AASMCs (**b**) co-cultured with AECs in hypoxia. The growth of PASMCs and AASMCs in 21% O_2_ was set as 100% and the proliferation of treated cells was calculated as a percentage of them. P = PASMCs, A = AASMCs, n = 8, Data are means ± S.D^. &^P < 0.05 vs. P (21%O_2_), **P < 0.01 or *P < 0.05 vs. P (5%O_2_) or A (5% O_2_), ^##^P < 0.01 vs. P + ATII or A + ATII. **B** NAC inhibited the constriction of PA and AA rings (**a**, **b**, **d**, **e**) the representative curve for the effect of NAC intervention on the constriction of PA and AA induced by AECs hypoxic culture medium (c, f) the summarized data of PA and AA rings maximum constriction. *AHCM* ATII hypoxic culture medium, n = 5, Data are means ± S.D. **P < 0.01 vs. AHCM

The concentration of H_2_O_2_ in AECs cell culture medium at 24 h under hypoxia was detected which was found to be within the range of promoting cell proliferation. The NAC intervention was performed in the co-culture system, which showed that both the proliferation of PASMCs (Fig. 10a) and AASMCs (Fig. 10a) were effectively inhibited by 10 mM NAC. These results indicated that H_2_O_2_ from ATII was involved in the proliferation of PASMCs and AASMCs. Besides, the concentration of H_2_O_2_ in AECs culture medium for 24 h under hypoxia was within the range of promoting constriction. Additionally, the constriction of PA (Fig. 10b) and AA rings (Fig. 10b) were effectively inhibited by 10 mM NAC. These results demonstrated that H_2_O_2_ from ATII was involved in the constriction of PA and AA.

## Discussion

It is well-known that pulmonary and systemic vessels respond differently to hypoxia and even more that isolated SMC can respond to hypoxia differently according to its origin [29]. Consistently, in this study we also confirmed that PA and RA respond differently to hypoxia and even more that PASMCs and AASMCs can respond to hypoxia differently. But we found that BA had the same response to hypoxia as PA in remodeling. Thus, we proposed the pulmonary vascular microenvironment created by AECs would be involved in the pulmonary vascular remodeling and constriction. In vitro, we showed that AECs not only promoted the proliferation of PASMCs, but also promoted the proliferation of AASMCs under hypoxia. And the hypoxic culture medium of AECs promoted the constriction of PA and AA rings. Furthermore, our data imply a role of regulation of AECs production of H_2_O_2_ depending on the pathway of ROS/ SOD2 as a possible mechanism of the regulation in the pulmonary vascular remodeling and constriction.

Some studies have confirmed that the response of the pulmonary vascular system to hypoxia is vasoconstriction rather than vasodilation in the systemic circulation [8, 30, 31]. Though BA belong to systemic circulation vessel, they are also exposed to AECs-related pulmonary vascular microenvironment. So in this study we found that PA and BA both show remodeling during alveolar hypoxia. A previous study comfirmed that alveolar hypoxia can cause hypoxic pulmonary vascular remodeling and vasoconstriction, and hypoxemia can not [30]. In the case of ventilatory dysfunction and low oxygen in the plateau, AECs primarily sense alveolar hypoxia and suffer the most dramatic changes in oxygen partial pressure. Therefore, we thought that AECs should play an important role in HPH. In our study, we observed the hemodynamic and morphological data of PA, BA and RA by constructing rat model of HPH. The results showed that hypoxia significantly increased RVSP, RV/ (LV + S) %, MT% and MA% of PA and BA, but had no significant effect on mCAP, MT% and MA% of RA. In addition, co-culture with AECs and treatment with AECs hypoxic culture medium not only significantly promoted PASMCs proliferation and PA constriction, but also significantly promoted AASMCs proliferation and AA constriction. Therefore, our data indicate that AECs play a key role in HPH and even more that the systemic vessel in the AECs-related microenvironment had also undergone remodeling and constriction.

It is interesting to consider the regulation of key antioxidant enzymes such as SOD and CAT in evaluating the role of redox-regulating signaling in pulmonary vascular diseases [32]. SOD can catalyze the rapid dismutation of O2^**·**^- to H_2_O_2_ and molecular oxygen, however, CAT can catalyzes the two-stage conversion of H_2_O_2_ to water and oxygen [32]. H_2_O_2_ is a stable and easily diffused out of the cell [18]. SOD has three isoforms, which are located in the cytosol (SOD1), mitochondria (SOD2) or extracellular compartment (SOD3), respectively [33]. Accumulating evidence has suggested that impaired activity of SOD2 and SOD3 contributed to pulmonary hypertension, and the level and activity of SOD1 has not been found significantly changed in human pulmonary hypertension [34, 35]. In this study, we found that hypoxia significantly increased the mRNA level of SOD2 and the content and activity of total SOD in AECs, while hypoxia has no significant effect on mRNA level of CAT. And the content of ROS and H_2_O_2_ in AECs and AECs culture medium also were increase under hypoxia. Hence, our data suggest that H_2_O_2_ derived from AECs can affect pulmonary vascular microenvironment and H_2_O_2_ may be caused by imbalance of SOD2 and CAT in AECs under hypoxia.

It has recently been reported that that H_2_O_2_ can regulate a variety of cellular functions, including proliferation, differentiation, and more generally gene expression [36, 37]. However, data on the basal concentrations of H_2_O_2_ and the thresholds for cell proliferation are limited. Studies have shown that treatment with a continuous extracellular source of H_2_O_2_ increases cell proliferation [26, 38]. Here, we determined the concentration range of H_2_O_2_ that promoted the proliferation of PASMCs and AASMCs. We found that the H_2_O_2_ produced by AECs cells was within the concentration range. Co-culture with AECs under hypoxic and the intermittent replacement of AECs hypoxic culture medium which were consistent with the conditions of continuous extracellular source of H_2_O_2_ can induce PASMCs and AASMCs proliferation. In other words, the method described above effected the proliferation of PASMCs and AASMCs by changing the microenvironment of PASMCs and AASMCs. In addition, NAC intervention reduced the proliferation PASMCs and AASMCs by co-culture with AECs under hypoxia. These results further indicate that H_2_O_2_ derived from AECs play an important role in PASMCs and AASMCs proliferation under hypoxia. Jin et al. [39] reported that exposure to H_2_O_2_ could cause smooth muscle constriction and dysfunction in isolated PA. Here, we also determined the concentration range of H_2_O_2_ that promoted the constriction of PA and AA. Similarly, the H_2_O_2_ produced by AECs during hypoxia was within the concentration range. The hypoxic culture medium of AECs promoted PA and AA rings constriction, and NAC intervention alleviated this effect. These results indicate that H_2_O_2_ derived from AECs plays an important role in the constriction of PA and AA. Taken together, our results could indicate that AECs are involved in remodeling and constricting of pulmonary vessels by releasing H_2_O_2_ under hypoxia and could explain the similar response of systemic arteries and AASMCs during treatment with AECs hypoxic culture medium and co-culture with AECs in vitro, respectively.

## Conclusion

The proliferation of PASMCs and the constriction of PA are critical to the development of HPH. Our study showed that the pulmonary vascular microenvironment was conducive to HPH, and AECs played a vital role in constructing the pulmonary vascular microenvironment. The continuous extracellular source of ROS is necessary for inducing PASMCs proliferation and PA constriction. H_2_O_2_ derived from AECs could affect the pulmonary vascular microenvironment and were involved in pulmonary vascular remodeling and constriction of HPH.

## Data Availability

We would like to share part of our data, because some of our data will be used in future research.

## Abbreviations

HPH: Hypoxic pulmonary hypertension
AECs: Alveolar epithelial cells
PA: Pulmonary artery
AA: Aortic artery
RA: Renal artery
PASMCs: Pulmonary artery smooth cells
AASMCs: Aortic artery smooth cells
mCAP: Mean carotid artery pressure
RV: Right ventricle
LV + S: Left ventricle plus septum
MT: Medial wall thickness
MA: Medial wall area
FBS: Fetal bovine serum
RVSP: Right ventricle peak systolic pressure
SOD: Superoxide dismutase
CAT: Catalase
ROS: Reactive oxygen species
H_2_O_2_: Hydrogen peroxide
NAC: N-acetylcysteine
CM: Culture medium
ANCM: ATII normoxic culture medium
AHCM: ATII hypoxic culture medium

## References

[1] Barbera JA, Blanco I (2009). Pulmonary hypertension in patients with chronic obstructive pulmonary disease: advances in pathophysiology and management. Drugs.

[2] Penaloza D, Arias-Stella J (2007). The heart and pulmonary circulation at high altitudes: healthy highlanders and chronic mountain sickness. Circulation.

[3] Wilkins MR, Ghofrani HA, Weissmann N, Aldashev A, Zhao L (2015). Pathophysiology and treatment of high-altitude pulmonary vascular disease. Circulation.

[4] Yan D, Li G, Zhang Y, Liu Y (2019). Angiotensin-converting enzyme 2 activation suppresses pulmonary vascular remodeling by inducing apoptosis through the Hippo signaling pathway in rats with pulmonary arterial hypertension. Clin Exp Hypertens.

[5] Liu M, Wang Y, Zheng L, Zheng W, Dong K, Chen S, Zhang B, Li Z (2014). Fasudil reversedMCT-inducedand chronic hypoxia-induced pulmonary hypertension by attenuating oxidative stress and inhibiting the expression of Trx1 and HIF-1alpha. Respir Physiol Neurobiol.

[6] Zakynthinos E, Daniil Z, Papanikolaou J, Makris D (2011). Pulmonary hypertension in COPD: pathophysiology and therapeutic targets. Curr Drug Targets.

[7] Wang LN, Yu WC, Du CH, Tong L, Cheng ZZ (2018). Hypoxia is involved in hypoxic pulmonary hypertension through inhibiting the activation of FGF2 by miR-203. Eur Rev Med Pharmacol Sci.

[8] Boger R, Hannemann J (2020). Dual role of the L-arginine-ADMA-NO pathway in systemic hypoxic vasodilation and pulmonary hypoxic vasoconstriction. Pulm Circ.

[9] Hough RF, Bhattacharya S, Bhattacharya J (2018). Crosstalk signaling between alveoli and capillaries. Pulm Circ.

[10] Signorelli S, Jennings P, Leonard MO, Pfaller W (2010). Differential effects of hypoxic stress in alveolar epithelial cells and microvascular endothelial cells. Cell Physiol Biochem.

[11] Makino A, Firth AL, Yuan JX (2011). Endothelial and smooth muscle cell ion channels in pulmonary vasoconstriction and vascular remodeling. Compr Physiol.

[12] Zhu P, Huang L, Ge X, Yan F, Wu R, Ao Q (2006). Transdifferentiation of pulmonary arteriolar endothelial cells into smooth muscle-like cells regulated by myocardin involved in hypoxia-induced pulmonary vascular remodelling. Int J Exp Pathol.

[13] Luo Y, Dong HY, Zhang B, Feng Z, Liu Y, Gao YQ, Dong MQ, Li ZC (2015). miR-29a-3p attenuates hypoxic pulmonary hypertension by inhibiting pulmonary adventitial fibroblast activation. Hypertension.

[14] Gore B, Izikki M, Mercier O, Dewachter L, Fadel E, Humbert M, Dartevelle P, Simonneau G, Naeije R, Lebrin F, Eddahibi S (2014). Key role of the endothelial TGF-beta/ALK1/endoglin signaling pathway in humans and rodents pulmonary hypertension. PLoS ONE.

[15] Grimmer B, Kuebler WM (1985). The endothelium in hypoxic pulmonary vasoconstriction. J Appl Physiol.

[16] Smith KA, Schumacker PT (2019). Sensors and signals: the role of reactive oxygen species in hypoxic pulmonary vasoconstriction. J Physiol.

[17] Bonnet S, Boucherat O (2018). The ROS controversy in hypoxic pulmonary hypertension revisited. Eur Respir J.

[18] Xu Z, Rothstein SJ (2018). ROS-Induced anthocyanin production provides feedback protection by scavenging ROS and maintaining photosynthetic capacity in Arabidopsis. Plant Signal Behav.

[19] Zuo L, Chuang CC, Clark AD, Garrison DE, Kuhlman JL, Sypert DC (2017). Reactive oxygen species in COPD-related vascular remodeling. Adv Exp Med Biol.

[20] Jaitovich A, Jourd'heuil D (2017). A brief overview of nitric oxide and reactive oxygen species signaling in hypoxia-induced pulmonary hypertension. Adv Exp Med Biol.

[21] Roberto D, Micucci P, Sebastian T, Graciela F, Anesini C (2010). Antioxidant activity of limonene on normal murine lymphocytes: relation to H2O2 modulation and cell proliferation. Basic Clin Pharmacol Toxicol.

[22] Hwang JW, Kim EK, Lee SJ, Kim YS, Moon SH, Jeon BT, Sung SH, Kim ET, Park PJ (2012). Antioxidant activity and protective effect of anthocyanin oligomers on H(2)O(2)-triggered G2/M arrest in retinal cells. J Agric Food Chem.

[23] Lo YC, Lin YC, Huang YF, Hsieh CP, Wu CC, Chang IL, Chen CL, Cheng CH, Chen HY (2017). Carnosol-induced ROS inhibits cell viability of human osteosarcoma by apoptosis and autophagy. Am J Chin Med.

[24] Costa RM, Filgueira FP, Tostes RC, Carvalho MH, Akamine EH, Lobato NS (2016). H_2_O_2_ generated from mitochondrial electron transport chain in thoracic perivascular adipose tissue is crucial for modulation of vascular smooth muscle contraction. Vascul Pharmacol.

[25] Li Z, Fang F, Xu F (2013). Effects of different states of oxidative stress on fetal rat alveolar type II epithelial cells in vitro and ROSinduced changes in Wnt signaling pathway expression. Mol Med Rep.

[26] Sigaud S, Evelson P, Gonzalez-Flecha B (2005). H_2_O_2_-induced proliferation of primary alveolar epithelial cells is mediated by MAP kinases. Antioxid Redox Signal.

[27] Wang YX, Liu ML, Zhang B, Fu EQ, Li ZC (2016). Fasudil alleviated hypoxia-induced pulmonary hypertension by stabilizing the expression of angiotensin-(1–7) in rats. Eur Rev Med Pharmacol Sci.

[28] Takano M, Nagahiro M, Yumoto R (2016). Transport Mechanism of Nicotine in Primary Cultured Alveolar Epithelial Cells. J Pharm Sci.

[29] Madden JA, Vadula MS, Kurup VP (1992). Effects of hypoxia and other vasoactive agents on pulmonary and cerebral artery smooth muscle cells. Am J Physiol.

[30] Hauge A (1969). Hypoxia and pulmonary vascular resistance. The relative effects of pulmonary arterial and alveolar PO2. Acta Physiol Scand.

[31] Lourenco AP, Fontoura D, Henriques-Coelho T, Leite-Moreira AF (2012). Current pathophysiological concepts and management of pulmonary hypertension. Int J Cardiol.

[32] Song T, Zheng YM, Wang YX (2017). Cross talk between mitochondrial reactive oxygen species and sarcoplasmic reticulum calcium in pulmonary arterial smooth muscle cells. Adv Exp Med Biol.

[33] McCord JM, Fridovich I (1969). Superoxide dismutase. An enzymic function for erythrocuprein (hemocuprein). J Biol Chem.

[34] Masri FA, Comhair SA, Dostanic-Larson I, Kaneko FT, Dweik RA, Arroliga AC, Erzurum SC (2008). Deficiency of lung antioxidants in idiopathic pulmonary arterial hypertension. Clin Transl Sci.

[35] Nozik-Grayck E, Woods C, Stearman RS, Venkataraman S, Ferguson BS, Swain K, Bowler RP, Geraci MW, Ihida-Stansbury K, Stenmark KR (2016). Histone deacetylation contributes to low extracellular superoxide dismutase expression in human idiopathic pulmonary arterial hypertension. Am J Physiol Lung Cell Mol Physiol.

[36] Hirobe T, Shibata T, Sato K (2016). Human fibroblasts treated with hydrogen peroxide stimulate human melanoblast proliferation and melanocyte differentiation, but inhibit melanocyte proliferation in serum-free co-culture system. J Dermatol Sci.

[37] Park WH (2016). Exogenous H_2_O_2_ induces growth inhibition and cell death of human pulmonary artery smooth muscle cells via glutathione depletion. Mol Med Rep.

[38] Porter KM, Kang BY, Adesina SE, Murphy TC, Hart CM, Sutliff RL (2014). Chronic hypoxia promotes pulmonary artery endothelial cell proliferation through H_2_O_2_-induced 5-lipoxygenase. PLoS ONE.

[39] Jin N, Rhoades RA (1997). Activation of tyrosine kinases in H_2_O_2_-induced contraction in pulmonaryartery. Am J Physiol.

